# Toward a Self-Updating Platform for Estimating Rates of Speciation and Migration, Ages, and Relationships of Taxa

**DOI:** 10.1093/sysbio/syw066

**Published:** 2016-08-19

**Authors:** Alexandre Antonelli, Hannes Hettling, Fabien L. Condamine, Karin Vos, R. Henrik Nilsson, Michael J. Sanderson, Hervé Sauquet, Ruud Scharn, Daniele Silvestro, Mats Töpel, Christine D. Bacon, Bengt Oxelman, Rutger A. Vos

**Affiliations:** 1 *Department of Biological and Environmental Sciences, University of Gothenburg, Box 461, SE-405 30 Göteborg, Sweden;*; 2 *Gothenburg Botanical Garden, Carl Skottsbergs Gata 22A, SE-41319 Göteborg, Sweden;*; 3 *Naturalis Biodiversity Center, Darwinweg 4, 2333 CR Leiden, The Netherlands;*; 4 *CNRS, UMR 5554 Institut des Sciences de l’Evolution (Université de Montpellier), Place Eugéne Bataillon, 34095 Montpellier, France;*; 5 *Department of Ecology and Evolutionary Biology, University of Arizona, 1041 E. Lowell, Tucson, AZ 85721, USA;*; 6 *Université Paris-Sud, Laboratoire Écologie, Systématique, Évolution, CNRS UMR 8079, 91405 Orsay, France;*; 7 *Department of Ecology and Evolution, University of Lausanne, 1015 Lausanne, Switzerland;*; 8 *Swedish Bioinformatics Infrastructure for Life Sciences, Department of Biological and Environmental Sciences, University of Gothenburg, Box 463, SE-405 30, Göteborg, Sweden;*; 9 *Department of Marine Sciences, University of Gothenburg, Box 460, SE-405 30 Göteborg, Sweden.*

**Keywords:** Bayesian phylogenetics, data mining, divide-and-conquer methods, GenBank, multilocus multispecies coalescent, next-generation sequencing, palms, primates, tree calibration

## Abstract

Rapidly growing biological data—including molecular sequences and fossils—hold an unprecedented potential to reveal how evolutionary processes generate and maintain biodiversity. However, researchers often have to develop their own idiosyncratic workflows to integrate and analyze these data for reconstructing time-calibrated phylogenies. In addition, divergence times estimated under different methods and assumptions, and based on data of various quality and reliability, should not be combined without proper correction. Here we introduce a modular framework termed SUPERSMART (Self-Updating Platform for Estimating Rates of Speciation and Migration, Ages, and Relationships of Taxa), and provide a proof of concept for dealing with the moving targets of evolutionary and biogeographical research. This framework assembles comprehensive data sets of molecular and fossil data for any taxa and infers dated phylogenies using robust species tree methods, also allowing for the inclusion of genomic data produced through next-generation sequencing techniques. We exemplify the application of our method by presenting phylogenetic and dating analyses for the mammal order Primates and for the plant family Arecaceae (palms). We believe that this framework will provide a valuable tool for a wide range of hypothesis-driven research questions in systematics, biogeography, and evolution. SUPERSMART will also accelerate the inference of a “Dated Tree of Life” where all node ages are directly comparable.

Many applications of phylogenetic trees in evolutionary and biogeographical research require, or strongly benefit from, the trees being as taxonomically complete as possible. In addition, phylogenetic inference itself also benefits from dense taxon sampling, for example, to break up long branches (Bergsten 2005). However, no method of phylogenetic inference can handle infinite amounts of data. Hence, there are trade-offs in the number of taxa and markers that can be usefully compiled into a data set. A major obstacle in selecting DNA sequence data for phylogenetic inference is that genetic sampling of species is taxonomically and geographically biased (Gotelli and Colwell 2001). A second hurdle is the fact that scientists have used different sets of genes and genetic markers for different taxa, both for intrinsic reasons (e.g., markers differ in information content among taxa, ease of amplification with Sanger sequencing or capture with hybrid enrichment techniques, and quality of source material) and because of lack of consensus on which markers to use for inferring phylogenies. For these reasons, compiling data sets for phylogenetics usually involves some combination of automated and manual data cleaning, data selection, and data integration. For example, to clean up candidate molecular data sets from GenBank (Clark et al. 2016) for phylogenetic inference, simple and automated rules can be applied to filter out short DNA sequences with little reciprocal overlap, sequences with significant amounts of missing data, sequences with poor taxonomic annotations (e.g., without full species names), and sequences that are unlikely to be true orthologs as determined by automated orthology assessment methods.

Several data cleaning and data selection pipelines have been developed to automate some of these steps for the purpose of generating suitable input data sets for phylogenetic inference. The PhyLoTA pipeline (http://phylota.nethttp://phylota.net, Sanderson 2008; Sanderson et al. 2008) pre-processes entire GenBank releases in pursuit of sufficiently overlapping reciprocal BLAST hits, which are then clustered into candidate data sets. This has the virtue that no assumptions are made about gene name annotations, but a drawback is that this “all-versus-all” approach is only computationally feasible for taxonomically constrained subsets of data from GenBank (e.g., only all sequences within a family or order). A more targeted approach is taken by the Phylogenetic Dataset Construction toolkit (PHLAWD, http://phlawd.net/) (Smith et al. 2009). This pipeline adds candidate sequences (identified by querying GenBank records for user-specified gene name annotations) to a user-provided set of seed sequences, provided that their reciprocal BLAST hit overlap is sufficient (this latter step is comparable to how PhyLoTA filters candidate cluster members). Although this results in data sets that are taxonomically broader than those obtained by PhyLoTA, a drawback is that under this approach only requested markers are collected—meaning that no unrequested regions are retrieved even if they contain phylogenetic information. Another drawback is that users need to specify all possible variations in the naming of gene regions. For instance, anyone who seeks to download 16S sequences from GenBank will encounter a near-endless array of orthographic and conceptual variations such as “16 S,” “16S,” “17S,” “SSU,” “ribosomal small subunit,” and “ribosomal small sub-unit” (note, however, that PHLAWD only uses gene names to locate candidate sequences, subsequently validating them by homology searches). Although workflows such as those implemented in PhyLoTA or PHLAWD are useful for assembling multiple sequence alignments, they do not by themselves create multilocus supermatrices with optimally broad taxon coverage.

## Identifiying and Applying Scalable Analytical Methods

**Figure 1.**
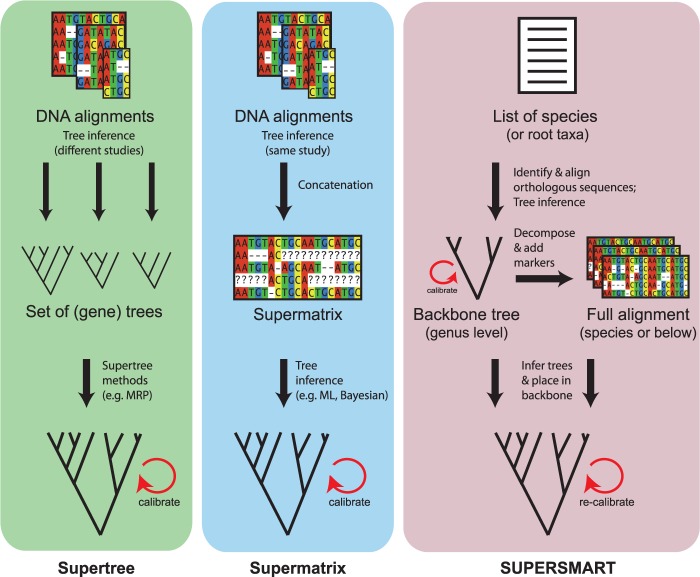
Methods for inferring large (dated) phylogenies. Schematic comparison of the supertree, supermatrix, and the SUPERSMART approaches.

Two main approaches have been developed to take advantage of the sequencing and phylogenetic efforts made so far, both of which have the capacity to handle very large numbers of terminal taxa: (i) *supertrees*, which involve the fusion of separate trees with at least some degree of taxonomic overlap, under parsimony, maximum likelihood, or Bayesian approaches (e.g., Bininda-Emonds et al. 1999; Nguyen et al. 2012, and references therein); and (ii) *supermatrices*, which are data sets containing sets of markers that share at least some taxa (de Queiroz and Gatesy 2007). Both approaches (see Fig. 1 for a comparison) present particular advantages as well as limitations (von Haeseler 2012), and alternatives are starting to emerge (Smith et al. 2013).

Supertrees are a common solution to produce a single, near-complete phylogenetic tree comprising all organisms in a clade. They can be built even when there is no genetic overlap among the subtrees they comprise (provided there is some taxonomic overlap), their inference is usually fast, and their mathematical properties well studied; these are factors that jointly have made supertrees (or variations thereof) the preferred choice for synthetic projects such as the Open Tree of Life (Hinchliff et al. 2015). However, one factor that has hampered the applicability of supertrees is the realization that just a small fraction of phylogenetic trees published can be retrieved through open data repositories or direct requests to authors of phylogenetic papers (Drew 2013; Stoltzfus et al. 2013).

Supermatrix approaches allow the estimation of large trees under a single analysis, relying directly on the underlying, primary data rather than on already defined tree topologies. Even a relatively small set of informative characters, such as a single gene or genetic marker scored across all taxa (or a small, well-connected set of markers), may potentially provide the backbone of a phylogeny and allow more rapidly evolving markers to resolve terminal relationships (Wiens 1998; Wiens 2006; Kupczok et al. 2010). A drawback with supermatrices spanning large taxonomic units and evolutionary times is homology assessment during the alignment of highly divergent or saturated sequences. Automated methods have been developed to detect rogue taxa (Aberer et al. 2013) and sequence saturation, and to perform profile alignment of very large supermatrices (Smith et al. 2009). Finally, a serious shortcoming of both supertree and supermatrix methods is that they typically assume that all data partitions are evolving according to the same tree, thus failing to account for processes such as incomplete lineage sorting, hybridization, and gene duplications/losses (Whidden et al. 2014). Polytomy resolvers are popular for enriching phylogenies with unsampled taxa, but can seriously distort downstream analyses (Rabosky 2015). It is thus clear that we need additional approaches that can handle vast amounts of data while applying robust methods for phylogenetic inference.

## Estimating Divergence Times

Considering the many methodological options available and the complexity of working with imperfect empirical data, it is not surprising that studies employing molecular dating analyses show a wide spectrum of results. This includes the various uses of available software, the varying quality and reliability of the fossils used (in terms of phylogenetic placement, absolute age, and proximity to the true timing of speciation of the taxon they represent), and the reliability of the molecular data supporting the chronograms (Sauquet et al. 2012). For instance, a phylogenetic study for genus $X$ that only uses a single fossil constraint $X_{1}$ for calibration of divergence times is likely to yield considerably younger ages as compared to the true divergences in the genus, if the fossil is considerably younger than the taxon it represents. If a similar study is done for the genus $Y$ (which happens to have a fossil record that closely matches the true time of origin of its taxon) and someone would like to produce a supertree of $X + Y$, the ages in the phylogeny would not follow the same absolute timeline. This incongruence could thus happen even if the supertree analysis is correctly performed and there are no uncertainties on the fossil placement. Although accuracy might increase through the use of both minimum and maximum age constraints in dating analyses, or more informative priors (Ho and Phillips 2009), based on these considerations few researchers would contest that estimated ages from different studies should not be compared or co-analyzed without proper correction. This implies that *dated phylogenies cannot be reliably “pasted together” in a similar way as traditional supertrees*. Moreover, it cautions against the use of dated phylogenies of various sources in meta-analyses, despite their potential as a powerful way of studying macroevolutionary processes, including the historical assembly of biomes (Crisp et al. 2009; Hoorn et al. 2010), dispersal across biotic barriers (Cody et al. 2010; Bacon et al. 2013; Bacon et al. 2015), or correlations between lineage age and diversity (Rabosky et al. 2012).

## Practical Impediments to Complex Analytical Workflows

The deluge of biological data has been followed by a corresponding, albeit more modest, growth in software development in ecology and evolution. This means that addressing relatively simple scientific questions may require researchers to master dozens of different analytical tools, often written in different programming languages and sometimes only available for certain operating systems or programming environments. The complexity of the task increases as each tool is constantly updated, improved, and made more complex, or superseded by better methods. To tackle this problem, there is an increasing tendency to create integrative analytical platforms for ecological and evolutionary research. This is seen in a number of popular software packages, for example, available in the R programming language (http://ropensci.org) and the Bio* toolkits in the Python, Ruby, Java, and Perl programming languages (http://open-bio.org), as well as stand-alone and online workflows (e.g., http://www.arborworkflows.com and http://www.biovel.eu). Since the choice of methodology will always depend on the research question, the nature of the data, and the researcher’s individual skills and knowledge to select and carry out analyses, any bioinformatic workflow to handle large amounts of data needs to be highly modular and flexible while retaining data standards to secure interoperability. Researchers should be allowed to make their own choices concerning, for instance, the inclusion/exclusion of taxa, the choice of genetic markers, which fossils and methodology to employ for molecular dating, the delimitation of areas for biogeographical and diversification analyses, and which analytical tools to use.

The fundamental but often neglected point of departure for any rigorous analysis should be that *all available data of adequate quality should be included*, provided that the computational methods used are scalable to this extent and unless there are specific reasons to warrant the exclusion of parts of the data. It is, in fact, hard to justify why an ecological, phylogenetic, or biogeographic study of a given group (e.g., family or order) should not include all high-quality sequences and fossil calibrations available for it. Free, online analytical platforms have provided an invaluable resource for the scientific community (e.g., the CIPRES gateway at http://www.phylo.org/portal2; Miller et al. 2015), but they most often require users to upload their own data for analysis, which is often a mere subset of the data potentially available. Modern biodiversity tools should thus tackle a moving target: the needs of addressing crucial ecological and evolutionary questions in the face of rapid *data growth and methodological development*.

## Presenting the Supersmart Approach

To address the challenges outlined above, we introduce a new conceptual and bioinformatic approach: SUPERSMART http://www.supersmart-project.org). SUPERSMART provides a platform for estimating time-calibrated molecular trees for potentially all sequenced eukaryote species, offering researchers a flexible, modular, integrative, and open-source platform for hypothesis-driven research in systematics, ecology, biogeography, and evolutionary biology. The package allows users to generate custom-made sets of robustly inferred, dated trees for further analyses, or to assemble aligned DNA data sets representing optimal combinations of sequenced genes/markers and taxa (see Fig. 1 for a comparison of SUPERSMART with supertree and supermatrix approaches).

### Overview

SUPERSMART is implemented as a modular framework making the bridge between the data handled (sequences, taxa, fossils, and trees), the records in a relational database that contains local copies of a number of public resources, and the operations needed to assemble these records into tailored data sets and analyze them. SUPERSMART is available as a virtual machine, and can be installed in environments that support the hosting and provisioning of free operating systems of the Linux family. Because SUPERSMART is delivered as a self-contained package with all necessary dependencies including nucleotide data, and each version is long-term stored and version-tracked, it also increases reproducibility of studies. The standard implementation runs through the command line, but a graphical user interface providing simpler functionality is also available through the Biodiversity Virtual e-Laboratory platform (http://biovel.eu).

**Figure 2.**
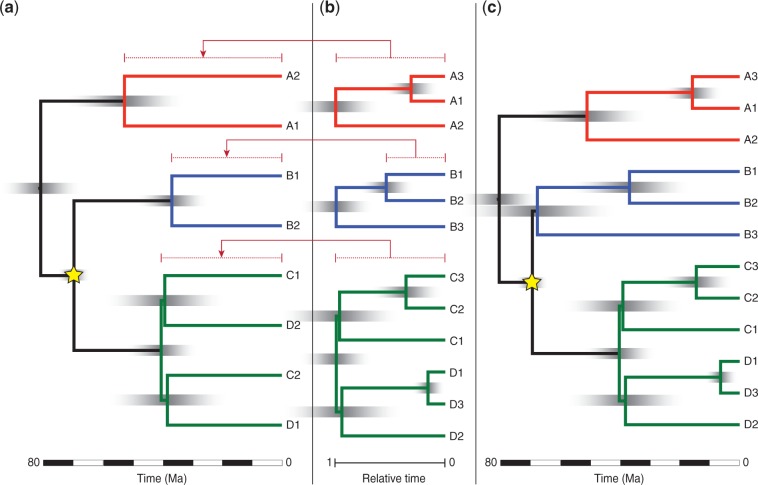
Basic overview of the three-step approach implemented in SUPERSMART. a) A backbone tree is inferred for four hypothetical genera (A, B, C, and D), each represented by two exemplar species. The backbone is calibrated using a fossil on the node indicated with a star (which may have an own confidence interval). In this example, two genera (C and D) appear in this analysis to be polyphyletic. b) The backbone tree is decomposed into three sets of taxa (red, blue and green) containing all the intrageneric taxa for which sufficient data are available. Genera C and D are merged into one taxon set because their exemplars were resolved as polyphyletic. Each taxon set is analyzed separately, yielding the trees shown. Hypothetical genus B shows that exemplars sometimes form an ingroup when more taxa are added; the pipeline attempts to minimize occurrences of this by picking exemplars with high sequence distance to one another. The clade trees have relative node ages and are scaled so that the most recent common ancestors of the respective exemplars have the same age as the equivalent nodes in the backbone (ages are indicated by the dotted lines). High posterior density intervals, indicated with gradients, are similarly scaled. c) The final tree is obtained by grafting. Note how the branch leading up to genus B is shortened to make room for B3, whose age has been scaled in proportion to the ratio of the ages of B1, B2 in the backbone and the clade tree. Note also how the highest posterior density (HPD) intervals have become proportionally larger, for example, on the root of genus B. The combined clade-level analysis resolved reciprocally monophyletic genera C and D without the use of constraints.

In its most common use, SUPERSMART will build arbitrarily sized, multilocus, recursive phylogenies for the group of interest (or one or more higher taxa) based on all suitable genetic markers. The included genetic markers may typically comprise DNA barcodes (Hebert et al. 2003), that is, COI, *rbcL*, *matK,* and ITS, as well as additionally selected markers that improve taxon coverage, including data generated with high throughput (next-generation) sequencing techniques. To enable the inclusion of a potentially very high number of taxa in the final results we employ a *three-step approach* (Fig. 2):

1. A backbone, higher level tree comprising a set of broadly sequenced exemplar species is initially built. This backbone tree (a phylogram) is time-calibrated using all suitable fossils from a fossil calibration table (see below for details).
2. The “backbone-chronogram” is decomposed into subclades (typically corresponding to families or genera) that are well supported and contain a manageable number of descendant species. All descendant taxa and suitable high-coverage genetic markers are added to these subclades. By default, SUPERMART includes one terminal per species, but users may choose to include all intra-specific taxa down to the level of individuals. Time-calibrated species trees are then inferred under the multispecies, multilocus coalescent model. The current implementation is done in *BEAST (Heled and Drummond 2010), but it could easily include other emerging methods such as STACEY for BEAST2 (Jones 2015). The resulting *BEAST trees have node ages representing relative divergence times from the most recent common ancestor of the corresponding exemplar species in the backbone tree. These trees are then re-calibrated (scaled) to the posterior age obtained for the clade in the first step to yield dated species-level trees.
3. The individual dated species-level trees are then grafted onto the backbone of the first step to obtain a complete species-level chronogram of directly comparable ages.

### Data Selection

SUPERSMART mines public databases for suitable DNA sequences by way of their globally unique taxonomic identifiers. Our present proof of concept adopts NCBI taxon IDs (Federhen 2012), but this could be extended to recognize other unambiguous identifiers, such as internet addresses or uniform resource identifiers. The user only needs to provide a list of taxa (species, genera, or higher taxonomic levels) to be included in the phylogeny. SUPERSMART expands any higher level taxon down to species level and maps all descendant species names onto unambiguous identifiers, by querying the TaxoSaurus service (http://taxosaurus.org) while taking into account synonyms and misspellings. TaxoSaurus follows the same synonyms as the NCBI taxonomy and it does some amount of fuzzy matching, but may occasionally return mismatches. Also, even when names match exactly they may not reflect the intent of the user: rare taxonomic homonyms do exist (e.g., across zoological and botanical nomenclature), and the NCBI taxonomy may recognize a different taxon concept—broader or narrower—than the user intended. The result of this name matching procedure is, therefore, written to a spreadsheet file available to the user for additional validation.

SUPERSMART then compiles candidate sets of DNA sequences for alignment, orthology assessment, and subsequent phylogenetic inference by querying a local, modified version of the PhyLoTA database. This database is the product of a workflow that crawls all taxonomically organized GenBank sequence divisions in the NCBI taxonomy, and performs all-versus-all similarity searches of the sequences subtended by each node. The sets of search hits are then grouped into single linkage clusters around a “seed sequence.” The sequences that are grouped in these clusters are generally an adequate starting point for phylogenetic inference, although several further data processing steps are necessary, as described below.

### Data Reduction

Many PhyLoTA clusters contain multiple sequences from the same taxon, often with extensive sampling bias toward “model organisms” (*sensu* PhyLoTA, i.e., very commonly sequenced organisms). As the standard goal of SUPERSMART is to infer species-level time-calibrated trees (although lower taxonomic levels are also supported), these clusters of sequences are reduced to more manageable data sets, containing approximately equal numbers of sequences for each species. The current approach is to select the most complete sequences, that is, the ones with the fewest DNA ambiguity symbols (Cornish-Bowden 1985) and that most closely approach the median length of all sequences for that species in that cluster. The goal is to remove short sequence fragments for markers for which longer sequences are available for a particular species. However, it is also best to avoid considerably longer stretches, which are likely to include fragments from other markers. Even though instances of either scenario are generally avoided due to the requirements that PhyLoTA imposes on overlap of reciprocal hits, these additional steps allow SUPERSMART to produce data sets that are more representative of intra-specific sequence variation and contain little missing data. Future versions may allow for other (or additional) selection criteria, such as the most recently deposited sequences, or accessions directly linked to publications.

### Data Merging

PhyLoTA clusters consist of sets of putatively homologous sequences grouped by taxonomic level (e.g., genera, families, or orders). The delimitation of clusters depends on the number of species included and the amount of available sequence data. Therefore, multiple “sister clusters” may exist for the same marker. For SUPERSMART to infer phylogenies that span several of these taxonomic levels, such putatively orthologous sister clusters need to be merged correctly. After evaluating several alternatives (such as using curated annotations of sequence metadata and searching for the protein translation of sequences to identify orthology), we concluded that running all-versus-all similarity searches was the best approach, since it could be applied also to noncoding regions and regions that lack standardized names.

### Multiple Sequence Alignment

Following these first steps, the DNA sequences stored in the SUPERSMART database are unaligned. As merged clusters can ultimately grow to very large numbers of sequences, we designed the pipeline in such a way that multiple sequence alignment takes place as a two-step process. First, the clusters as assigned by PhyLoTA are aligned (after data reduction). Many programs for multiple sequence alignment exist and can potentially be used by SUPERSMART; wrappers for several of them are provided. By default the pipeline uses MAFFT (Katoh and Standley 2013), which gives good results on a variety of different markers and has the virtue of being able to auto-select its alignment strategy given the data—something that comes in handy for SUPERSMART. Second, orthology among clusters at taxonomic levels higher than PhyLoTA can manage is assessed using the all-versus-all approach described above, but applied only to the seed sequences around which the candidate clusters were built. Finally, orthologous “sister clusters” are merged by profile alignment, which is a less computationally intensive procedure than multiple sequence alignment, involving the reconciliation of blocks of previously aligned sequences. By default, the profile alignment step uses MUSCLE (Edgar 2004).

### Marker and Taxon Selection

The data selection steps outlined above provide a wealth of species-level multiple sequence alignments, although not all of them may be equally well suited for inferring a backbone tree. An optimal balance must be found between taxon sampling, taxon overlap, sequence divergence, and overall size (and sparseness) of the combined data. In our multistep approach, this optimum is further influenced by which exemplar species are selected for the backbone inference. Our solution is to first filter out all taxa that share too few markers with other taxa, both inside their own genus and in relation to other genera. Subsequently we select, for each genus to be represented, the two species that most frequently form the most distal pair when computing all pairwise sequence distances within the genus. This is done iteratively for each candidate alignment. During this step we weight the occurrence of distal pairs by $n -1$, where $n$ is the number of pairwise comparisons within each alignment. The rationale is that the most distal pair among a large number of comparisons is more likely to “cross the root” of the containing genus (or at least, represent a deep split) than in smaller alignments.

**Figure 3.**
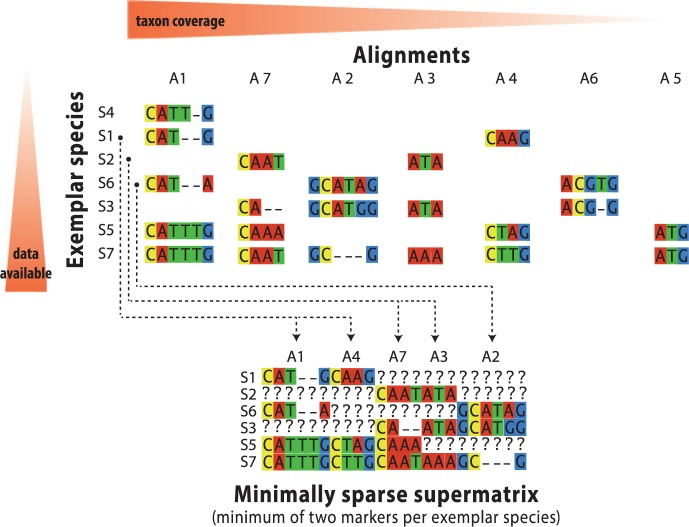
Illustration of the classic knapsack problem applied to the optimal choice of species and alignments (markers) for compiling DNA alignments. Seven exemplar species (S1–S7) are put in ascending order by their occurrence in the candidate alignments (A1–A7) which are in turn ordered by taxon coverage. In this example, the minimum number of alignments per species is set to two. The supermatrix is then compiled as described in the text. The resulting matrix consists of five alignments and only six species, since the number of alignments in which species S4 occurs does not meet the required minimum.

Once all exemplar species are identified, candidate alignments are selected for concatenation as input to the backbone analysis. For this step the user can choose to define a maximum amount of average pairwise sequence divergence (to prevent the inclusion of saturated alignments) and a minimum and maximum number of alignments within which each species must occur. SUPERSMART then attempts to tackle the “*knapsack problem*” (Martello and Toth 1990) of packing the required number of suitable alignments into a minimally sparse supermatrix. The greedy approximation approach we take (Fig. 3) is to sort the exemplar species in increasing order of participation in candidate alignments (i.e., rarely sequenced species are treated first). We then sort the alignments by decreasing taxon coverage. Finally, we iteratively visit the species, and for each of them we add its available alignments to the supermatrix, until the focal species’ maximum participation threshold has been reached or no further alignments are available.

### Phylogenetic Reconstruction of the Backbone

Using the supermatrix of concatenated alignments for the exemplar species, we then infer a backbone phylogeny. Given that the supermatrix may span several thousands of taxa, we employ highly scalable tree inference methods, providing end users with a choice between ExaML (Stamatakis and Aberer 2013), RAxML (Stamatakis 2014) and PhyML (Guindon et al. 2010), which are based on maximum likelihood algorithms, and ExaBayes (Aberer et al. 2014) based on Bayesian inference.

### Time Calibration Using Fossils

SUPERSMART maps all suitable user-provided fossil records belonging to the focal clade onto the backbone trees inferred in the previous step. The trees are then dated using the relaxed clock algorithm Penalized Likelihood (Sanderson 2002), as further developed and implemented in treePL (Smith and O’Meara 2012), which can handle very large numbers of terminals. This step inputs samples of trees to produce confidence intervals of node ages rather than point estimates. In the future, SUPERSMART will take advantage of the Fossil Calibration Database (Ksepka et al. 2015) for automated fossil retrieval to calibrate nodes on backbone trees.

### Species-Level Analyses

Using a consensus of the backbone topologies, SUPERSMART assesses whether genera (represented by the exemplar species) are monophyletic. If this is not the case, it traverses up the backbone until it finds a higher level monophyletic group. For each of the clades selected, all available alignments are then compiled. The set of alignments selected for the focal clade is then analyzed under the multispecies, multilocus coalescent implemented in *BEAST (Heled and Drummond 2010). Other tree inference methods could be easily implemented in future versions, for instance, computationally less expensive species-tree methods such as ASTRAL (Mirarab et al. 2014).

All the resulting ultrametric species-level subtrees are then grafted back onto the backbone chronogram (Fig. 2). First, all branch lengths on each subtree are rescaled such that the most recent common ancestor of the exemplars in the subtree is set to the same age (distance to the tips) as the equivalent node in the backbone tree. As both trees are ultrametric and generated under the same time frame, this distance can be directly transferred. If the exemplar species in the subtree are on either side of the root, then the pair of exemplars in the backbone can simply be replaced by the subtree. If not, then the distance between the most recent common ancestor of the exemplars in the subtree and the root of the subtree is computed. This difference is finally subtracted from the branch leading up to the exemplar pair in the backbone, and from that point onwards the subtree is grafted in place of the exemplar pair. The result is a time-calibrated species-level phylogeny with directly comparable clade ages, including all suitable species and genetic markers publicly available, and any additional genetic or fossil data provided by the user.

### Macroevolution

The species-level, dated phylogenies produced with SUPERSMART can be immediately used for various phylogenetic and biogeographical analyses. These include inferences of, for example, migration, diversification, and niche evolution, some of which are already integrated with SUPERSMART at http://www.biovel.eu.

## Empirical Examples

We present the functionality of SUPERSMART in its current implementation using two empirical data sets: the mammalian order Primates (primates; including lemurs, lorises, tarsiers, monkeys, and apes) and the plant family Arecaceae (palms). These taxa provide contrasting examples commonly encountered in eco-evolutionary research. Primates have been extensively studied by the scientific community, leading to a massive accumulation of DNA sequences, which are, however, highly biased toward our own species, near relatives, and model organisms such as the *Rhesus* monkey. Even so, the estimated number of living species ranges from 249 (http://www.catalogueoflife.org) to 376–450. This shows how the classification of even such a well-studied clade remains a topic of debate, which can have a substantial impact on downstream analyses such as the estimation of diversification rates (Faurby et al. 2016b). We also use the Primates to explore what effect different combinations of parameter settings have on supermatrix assembly, and on the robustness of subsequent phylogenetic inferences. In contrast, palms comprise a much larger number of extant species (2561 according to Catalogue of Life) and several economically important genera such as the coconut (*Cocos*), date (*Phoenix*), and oil palm (*Elaeis*), but have received less attention and are, therefore, the subject of much lower taxonomic and genetic coverage in public sequence databases. The palms are also used here to highlight the use of SUPERSMART in historical biogeography. Table 1 summarizes the statistics on both primates and palms included in the SUPERSMART analyses.

**Table 1:** Summary statistics for SUPERSMART runs for the order Primates and the family Arecaceae

	Primates	Arecaceae
Genera included in analysis	71	197$^{\mathrm{a}}$
Species included in analysis	363	1112$^{\mathrm{a}}$
Terminals (backbone tree / species tree)	117 / 251	293 / 733
Average posterior support (backbone tree / species tree)	0.950 / 0.681	0.868 / 0.351
Number of loci (backbone tree / species tree)	65 / 108	26 / 37
Total base pairs in mined sequence data	${\sim}$16 million	${\sim}$6 million
Min–max length of alignments (base pairs)	46–7360	87–6870
Calibrations	8	6
Subclades	31	29

^a^Including three outgroup genera with 34 species.

### Primates

To illustrate the trade-offs imposed by various parameters that influence the data selection process in primates, we explored their parameter space. We tested the effect of imposing different thresholds of the minimum number of markers necessary for a species to be included in the analysis (Supplemetary Fig. S1 and Supplementary Material available on Dryad at http://dx.doi.org/10.5061/dryad.sk81k). As expected, as more markers are required per taxon, the number of taxa decreases rapidly. While here we show what happens when *all* taxa must meet the same threshold (Supplemetary Fig. S1 available on Dryad), a more pragmatic approach is to set the minimum number of markers to a low value, but allow the maximum number to be higher, so that at least some—if not all—included taxa will have greater marker coverage. We have found that this approach increases the density of the “marker graph” (a network in which taxa are connected if they share a certain marker), reducing both its modularity and the average path length between taxa. We also explored how the minimum number of required markers and their maximum amount of divergence affect the posterior probability of nodes (Supplementary Fig. S2 available on Dryad). The average posteriors in this example are largely a function of the number of markers. Even at high levels of allowed divergence (up to and beyond 20%) we find no evidence of deteriorating results (e.g., due to saturation), whereas these settings allow for the acceptance of more candidate markers, which may be important in poorly sequenced groups.

**Figure 4.**
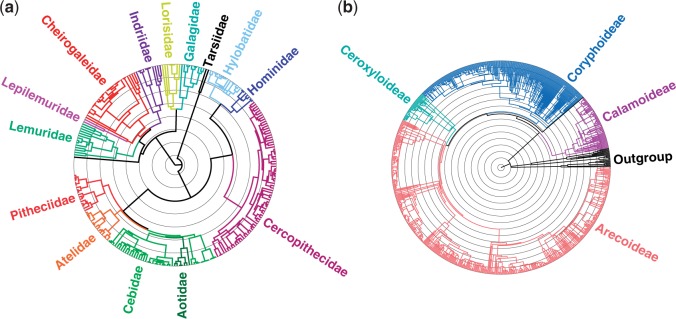
Time-calibrated phylogenies of (a) the mammal order Primates (primates) and (b) the plant family Arecaceae (palms) inferred using SUPERSMART. The families in (a) and the subfamilies in (b) are outlined. Internal concentric circles represent 10 myr bins. See Supplementary Figures S3 and S4 available on Dryad for fully annotated trees.

Figure 4a shows the simplified results from the analysis of primates, whereas Supplementary Figure S3 available on Dryad presents the fully annotated species tree. The species tree of the Primates comprise 251 species, calibrated using the same fossils as in Vos and Mooers (2004). The inferred topology of the extant clades Strepsirrhini (crown age ${\sim}$53 million of years, Ma), tarsiers (${\sim}$20 Ma), New World monkeys (${\sim}$26 Ma), Old World monkeys, and apes (${\sim}$20 Ma) is congruent with the generally accepted understanding of primate systematics and approximates the dating of events (e.g., basal diversification preceding the K–Pg boundary by ${\sim}$15–20 Ma) as previously reported (Bininda-Emonds et al. 2007; Springer et al. 2012). The precise relationships among the families within the New World monkeys (Platyrrhini) remain a matter of debate (Opazo et al. 2006), but all genera are supported here as monophyletic. Our results suggest an initial split of the family Pitheciidae and a close relationship between the families Atelidae and Cebidae. The Old World monkeys (Cercopithecidae) comprise the two monophyletic subfamilies Colobinae and Cercopithecinae$,$ which is in agreement with a previously published primate supertree (Vos 2006). Hominoidea are well resolved and strongly supported, including the resolution of the hominoids. The phylogeny is based on more than 100 markers, of which each species was required to have at least three to be included in the backbone analysis. Relatively fewer sequences were available for the inference of Tarsiiformes and Strepsirrhini. Strepsirrhini split into one Malagasy and one non-Malagasy clade. The lemurs, native to Madagascar, are represented by four families that are well resolved in our tree. Only in a single case—the genus *Hylobates*—did the clade-level multispecies multilocus coalescent analysis using *BEAST recover a deeper root than what was compatible with the backbone tree.

### Palms

The palm phylogeny was calibrated with the macrofossils $\dagger$*Hyphaene kapelmanii*, *Mauritiidites crassibaculatus*, *Sabalites carolinensis*, and *Tripylocarpa aestuaria* (Harley 2006) using topological placements based on morphological synapomorphies (Supplementary Table S1 available on Dryad). In addition, we included a well-preserved flower in Dominican amber ($\dagger$*Socratea brownii*) (Poinar 2002), which has been used to constrain the crown node of extant *Socratea* due to its sessile, staminate flowers with high stamen number (20–100), which are diagnostic floral characters found among extant species in the genus (Bacon et al. 2016). An exponential prior was applied, with mean $=$ 22.5 Ma and standard deviation $=$ 1.17.

The phylogenetic analysis recovered highly supported subfamilial relationships that are consistent with previous studies (Baker et al. 2009) and the current understanding of the morphological evolution in the group (Dransfield et al. 2008) (Fig. 4b and Supplementary Fig. S4 available on Dryad). Most of the major tribes and genera are also resolved as monophyletic with strong support, except for subfamily Coryphoideae and its members that are inferred as a polytomy. The mean crown age of the family is younger (85.8 Ma; Supplementary Table S2 available on Dryad) compared to one previous analysis estimating it to ca. 100 Ma (Couvreur et al. 2011). The crown nodes of each of the subfamilies differ between the SUPERSMART and earlier results, some being older and others younger than previous estimates (Supplementary Table S2 available on Dryad). We also compared our tree topology with a recently published palm phylogeny (Faurby et al. 2016a), finding both similarities and differences (Supplementary Fig. S5 available on Dryad) that likely reflect differences in the underlying data and analytical steps between our studies.

We then performed a biogeographic analysis of palms using our new dated tree. First, we downloaded all geo-referenced species occurrences for palms from GBIF (comprising 724,002 records; downloaded on 1 April 2016; http://doi.org/10.15468/dl.2083tb). Then, we performed automated data cleaning steps as implemented in the function GeoClean of the package speciesgeocodeR (Zizka and Antonelli 2015; Töpel et al., in press). The resulting data set was used as input for a bioregionalisation analysis in Infomap Bioregions (Edler et al., this issue), with minimum and maximum cell sizes ranging between 2${}^\circ$ and 4${}^\circ$, and minimum and maximum cell capacity ranging between 10 and 100 records per cell. We then coded the presence or abscence of all species in the palm phylogeny in each of the bioregions identified using SpeciesGeoCoder (Töpel et al., in press) and estimated ancestral ranges using the dispersal–extinction–cladogeneis (DEC) model (Ree and Smith 2008) under an unconstrained scenario (i.e., no time stratification or arbitrarily defined dispersal matrices) in the R-package BioGeoBEARS (Matzke 2013). We calculated the number of dispersal events through time between all pairs of areas (i.e., when a lineage disperses, or expands, from area $a$ to area $b$ and vice versa). Number of dispersal events were computed for time bins of 5 Ma across the duration of the whole tree. Under the DEC model, dispersal events take place along branches. As branches can fall into more than one 5 Ma time bin, we weighted the dispersal event relative to the length of that branch that fell in the bin (e.g., if half the branch fell in the bin, we counted half an event). Because phylogenies have an increasing number of lineages toward the present, more lineages can potentially disperse into a different area. To account for this, we also computed relative numbers of dispersals by dividing the number of dispersals by the total branch length per time bin (Antonelli et al. 2015) (Supplementary Fig. S6 available on Dryad).

**Figure 5.**
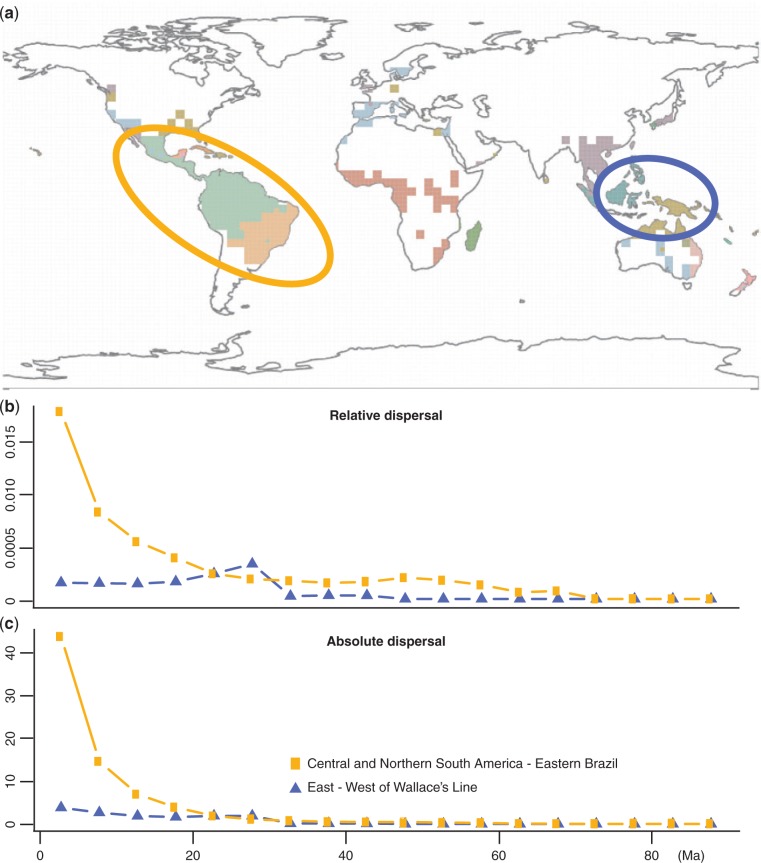
Results from the biogeographic analyses of palms. a) Bioregionalisation analysis based on ca. 724,000 species occurrence records, highlighting the two regions analyzed below. b) Relative number of dispersal events (or range expansions) in proportion to the number of lineages in the phylogeny in which such events could have taken place between Northern South America and Central America (as one area) and eastern South America, and between east and west of Wallace’s Line. c) A similar analysis as in (b), but showing the absolute number of events. See text for details on the analysis.

The bioregionalisation analysis identified an optimum of 19 bioregions of palms, reflecting major species assemblages among and within continents (Fig. 5a). Our biogeographical analysis resolved an origin of the palm family in Central and South America (Supplementary Fig. S7 available on Dryad), with dispersal out of the Americas occurring only around 70 Ma. This result in consistent with early hypotheses of palm evolution (Moore 1973), but contrasts with more recent studies that suggest a Laurasian (North America and Eurasia) origin of the family (Couvreur et al. 2011; Baker and Couvreur 2013). We show the results from the dispersal through time analyses for two sets of areas: between Central and Northern South America and Eastern Brazil, and across Wallace’s Line (Fig. 5b–c). We identified a total of 75 dispersals (or range expansions) between Central and South America and Eastern Brazil throughout the evolutionary history of palms, with a marked increase in absolute and relative dispersal rates over the last 20 Ma and accentuated in the last 10 Ma. This most recent increase might reflect the retraction of the Miocene megawetland, called the Pebas System, that covered a large portion of western Amazonia until ca. 10 Ma and led to the subsequent expansion of lowland tropical forests (Hoorn et al. 2010), the primary habitat of palm species (Couvreur et al. 2011). Biotic interchange across Wallace’s Line (estimated from 14 dispersal events) show a 2-fold increase in relative dispersal across the region at ca. 28 Ma, consistent with the reorganization of tectonic plates in the region between 30 and 20 Ma, which facilitated biotic dispersal in the region (Hall 1998).

## Validation of the Multistep Tree Inference

To assess the performance and accuracy of our two-step phylogenetic inference approach we conducted a study using simulated sequence data, and we added functionality to the SUPERSMART platform to replicate empirical data sets generated by simulation. For the results we discuss below, we aimed at producing a synthetic data set that resembles the sequence data for the Primates example, obtained with the data mining features in SUPERSMART, both in terms of the sequences themselves (e.g., divergence and indels) and in terms of biases in taxon sampling. The synthetic data set consists of a simulated phylogeny and a set of molecular sequences that are simulated to have evolved according to the tree. The sequences were then used as input for SUPERSMART and other commonly used phylogenetic tools with the aim of re-estimating topology and divergence times of the simulated tree. We then assessed the differences in topology and node ages between the simulated tree and the inferred one for each tree inference method.

### Simulation of Synthetic Data Sets

**Figure 6.**
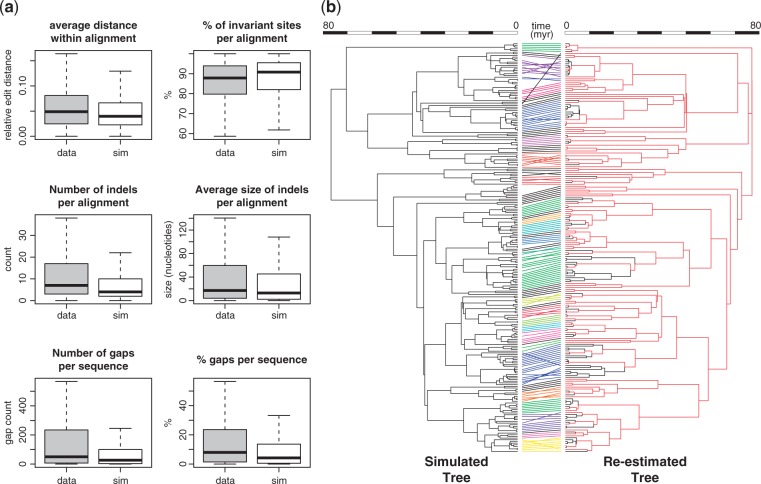
Validation of the three-step phylogenetic inference process. a) Comparison of the molecular data for the primate tree inference and the replicated data set obtained from sequence simulations. Boxes show the interquartile range of each property for all alignments and its median as a black line. The ends of whiskers represent the lowest and highest value within 1.5 times the interquartile range of the lower and upper quartile, respectively. Gray and white boxes show real and simulated data, respectively. (b) Simulated tree (left) matched with the tree that was re-estimated from the synthetic data set using SUPERSMART. Species present in both trees are connected by lines which are color coded by the subclades that the backbone tree was decomposed into. Branches in the re-estimated tree that form the backbone are colored in red. A comparison of fully annotated trees is shown in Supplementary Figure S8 available on Dryad.

To obtain a known “generating” tree we simulated a phylogeny that resembles the primate phylogeny obtained with SUPERSMART with regard to its size, depth, number and size of genera, and parameter values of the birth–death process. We estimated the parameter values for the latter by fitting a birth–death model using maximum likelihood, as implemented in the R-package ape (Paradis et al. 2004) to the empirical primate phylogeny. Tips of the simulated tree were then assigned to genera approximating the size and age distribution of genera in the original tree. In this way, genera were kept monophyletic while being shuffled in the replicated phylogeny. Next, we replicated the set of orthologous sequence clusters obtained from the multiple sequence alignments in the original analysis. To this end we selected substitution models and their parameter values using the R-package phangorn (Schliep 2011) on the empirical data sets and applied these when simulating sequences on the simulated tree using the R-package phylosim (Sipos et al. 2011) Simulated alignments that were invariable were removed from the data set. Basic properties of the simulated tree set of alignments are similar to the actual data (Fig. 6). Replication of molecular sequence data sets is implemented in SUPERSMART to provide users with the possibility of validating custom analyses. All code and results of the simulation study are available in the Supplementary Material on Dryad.

### Tree Inference from the Synthetic Data set

The synthetic sequence data set was used as a starting point for phylogenetic inference with SUPERSMART, using ExaBayes and *BEAST for backbone- and subclade inference, respectively. We used the same pipeline settings (e.g., number of markers in backbone and clade matrices, number of generations for the Bayesian backbone- and clade tree inference, maximal allowed distance within an alignment etc.). A comparison of the simulated “generating” tree and the tree that was re-estimated from the synthetic data (Fig. 6b; a fully annotated tree is provided in Supplementary Fig. S8 available on Dryad) shows that both tree topology and node ages agree well. Branch lengths in the re-estimated tree appear to be slightly overestimated, possibly because the re-estimated tree was calibrated using TreePL, which systematically overestimate node ages (Sanderson and Doyle 2001).

To assess the performance of the two-step approach implemented in SUPERSMART compared to other software tools, we compiled matrices from the synthetic data set and used these as input for maximum likelihood inference with the well-established tools ExaML (Stamatakis and Aberer 2013) RAxML (Stamatakis 2014) and GARLI (Zwickl 2006). We restricted our comparison to these methods because state-of-the-art Bayesian methods become prohibitive for the dimension of our data set (228 taxa and 289,978 sites). Each tool could, therefore, only be tested with a relatively low number of bootstrap replicates of the input matrix. We quantified the differences in topology between the synthetic generating tree and the inferred trees using the Robinson–Foulds metric, which counts the number of bipartitions that occur in one tree but not in the other, and vice versa. Differences in divergence times between two trees were calculated as the squared Euclidean distance between the branch lengths for each bipartition, as described by Kuhner and Felsenstein (1994). All distances were normalized by dividing by the total number of bipartitions.

The normalized Robinson–Foulds distance between the synthetic tree and the tree re-estimated with SUPERSMART was 0.31 (ExaML: 0.36; RAxML: 0.38; GARLI: 0.37). The normalized squared Euclidean distance between the synthetic tree and the SUPERSMART tree was 43.82 (ExaML: 59.61; RAxML: 55.39; GARLI: 55.27), meaning that on average, the disagreement between branch lengths was about 6.6 myr. Note that the squared Euclidean distance also includes branch lengths for bipartitions that are not shared by both trees.

It is important to mention that it would in general be possible to further optimize the workflows for inferences with ExaML, RAxML, and GARLI to obtain trees that are more similar to the synthetic tree. However, given the size of the data set, further fine-tuning of parameters would require immense capacities in CPU and memory. Our simulation study shows that SUPERSMART is able to estimate topology and divergence dates in a simulated phylogeny with a good degree of accuracy (Fig. 6b) and that the performance is comparable to other state-of-the-art phylogenetic inference software. We therefore argue that the three-step approach implemented in SUPERSMART is fruitful and viable for inferring large phylogenies in an entirely Bayesian framework, and capable of accommodating vast amounts of genetic data.

## Interactions with Other Initiatives

SUPERSMART is designed as a community-based platform that will complement and interact (rather than compete) with many ongoing initiatives worldwide. Supplementary Figure S9 available on Dryad outlines some of the anticipated interactions and data exchange possible during different operational levels. For instance, a related application which efficiently assembles sequence data for a prespecified list of target species is PHLAWD, as used in Zanne et al. (2014). SUPERSMART has similar goals but differs from PHLAWD by dealing more extensively with name resolution, homology/orthology assessment, simultaneously optimizing taxonomic and genetic coverage, time calibration through a curated fossil database, and the extensive support for, and use of, plug-in tools. Two other recent projects have similarities with SUPERSMART, although their scope is more limited. PUmPER (Izquierdo-Carrasco et al. 2014) assembles multiple sequence alignments for a given group in the NCBI taxonomy, but it infers maximum likelihood gene trees, omitting the Bayesian multispecies, multilocus coalescent approach taken by SUPERSMART, the time calibration (dating) step, and taxonomic name resolution. In addition, the PUmPER approach is designed to infer a tree in one single step, which may pose scalability problems for very large numbers of taxa, as opposed to the recursive approach we present here. Huerta-Cepas et al. (2014) introduce such a recursive approach through their nested phylogenetic reconstruction methodology. Crucially, in both cases, the resulting estimate of phylogeny is a maximum likelihood gene tree whose branch lengths are not proportional to time, which hampers their application in most diversification and biogeographical analyses.

## Prospects, Weaknesses, and Limitations

The version of SUPERSMART released with this publication contains a fully functional set of tools for producing time-calibrated species phylogenies from input lists of taxa. SUPERSMART is a key component in the “comparative biogeography” framework (Antonelli Forthcoming), being readily linked to data-driven identification and delimitation of biogeographical regions (Edler et al., this volume); coding of species into discrete spatial units for ancestral range analyses (Zizka and Antonelli 2015; Töpel et al., this issue); the estimation of rates of migration, speciation, and extinction; extracting subtrees from global species-level chronograms (http://phylotastic.org); among numerous other macroevolutionary and biogeographical analyses, some of which are exemplified here for the palms.

Our approach for inferring phylogenies first produces a backbone topology of exemplar taxa, decomposes it, and then grafts species trees with more dense taxon sampling onto it (Fig. 2). Although this is key to the workflow’s ability to deal with vast amounts of data, this approach has potential weaknesses. The ages of the species trees that are grafted onto the backbone topology are usually based on secondary calibration points (unless fossils are available for the root of the species tree). This may be viewed as problematic both on purely philosophical grounds, in that error in the age estimates of the backbone will propagate and may be compounded, as well as on more empirical grounds because less dense taxon sampling (as is the case for the backbone tree) may bias age estimates for nodes. Given fewer taxa, fewer substitutions will be reconstructed in saturated markers and so node ages may be reconstructed as too recent. Likewise, lineage-specific rate variation among backbone exemplars may introduce artifacts in node age estimates. It is, therefore, crucial to continue exploring the impact of secondary calibrations on divergence time estimation, and develop methods to properly model this approach (Schenk 2016). In addition, the selection of exemplar taxa whose most recent common ancestor does not coincide with the clade root, for example, as in the hypothetical case of grafting clade B onto the backbone in Figure 2, can result in a rescaled root node age that is older than its parent node, thereby resulting in a negative branch length. These weaknesses can be addressed to some extent by users, by carefully selecting the number of exemplars to include in the backbone, the inference tool that is used, and the sequence divergence thresholds.

Another source of potential error lies not in the backbone topology but in the species tree. SUPERSMART uses *BEAST to infer species trees. This approach requires careful monitoring to ensure that effective sample sizes for salient parameters are suffi-ciently large; users must, therefore, allow for enough MCMC generations to accomplish this. In addition, users should understand what *BEAST does and how it is parameterized: SUPERSMART generates input files for *BEAST that specify generally reasonable settings for a multispecies, multilocus coalescent analysis, but taxon-specific settings may very well be improved upon (which at time of writing means that users need to edit BEAST XML files by hand, as the graphical tool BEAUti does not load previously generated input files). The potential caveats of SUPERSMART mean that, like all methods for phylogenetic inference, users should understand what they are doing and carefully evaluate all their results. The large number of ways in which methodological artifacts can be introduced along the way of a complex phylogenetic analysis means that “black boxes” that produce credible results without trial and error are not likely to become available any time soon, and SUPERSMART is no exception to this.

Finally, a major limitation of SUPERSMART is computational scalability. With the wealth of genomic data being produced, a next step in development will be to produce backbone trees using full genomes and coalescent models. Another challenge will be to make PhyLoTa updates fully automated, so that GenBank is downloaded and its sequences clustered several times a year. Several additional enhancements are planned and will be added successively. Anyone can join the users’ list and request additional features, and those wishing to contribute to the code and project are invited to join the developers’ list at http://www.supersmart-project.org.

## Conclusion

Phylogenetic research has arguably never been as exciting—but also as challenging—as today. We have entered the era of big data and cannot ignore its potential impact on the evolutionary, biogeographical, and ecological questions we address. Integrative bioinformatic solutions such as SUPERSMART will aid researchers in several disciplines to tackle the “moving target” of data accumulation, methodological development, and theoretical advances.

## Availability

All source code for this project is freely accessible under an MIT license at http://www.supersmart-project.org, where tutorials, example files, and other relevant information are continuously updated and improved.

## Supplementary Material

Data available from the Dryad Digital Repository: http://dx.doi.org/10.5061/dryad.sk81k.

## Funding

This work was supported by the Swedish Research Council [B0569601 to A.A.] and [2012-3917 to B.O.]; the European Research Council under the European Union’s Seventh Framework Programme [FP/2007-2013, ERC Grant Agreement No. 331024] and a Wallenberg Academy Fellowship [to A.A.]; project BioVeL [Grant No. 283359 to H.H.]; Carl Tryggers stiftelse [CTS 12:24, 11:479 and 12:507 to F.L.C., D.S., and M.T.]; Wenner-Gren [to D.S.]; and FORMAS [215-2011-498 to R.H.N.].
